# Supplementary material for: A Low FODMAP Diet Is Nutritionally Adequate and Therapeutically Efficacious in Community Dwelling Older Adults with Chronic Diarrhoea
Source: Nutrients. 2020 Sep 30;12(10):3002. doi: 10.3390/nu12103002 (PMC7600506; doi:10.3390/nu12103002)
Supplement: Supplementary file 1 [file nutrients-12-03002-s001.pdf]

Supplementary material

Supplementary Table 1. Changes of participant scores from baseline scores to follow up scores for each SAGIS domain and question with percentage of participants that experienced worsening of symptom's, no change of symptoms and an improvement of symptoms.

SAGIS subdomains and individual questions	Pre scores No problem /Mild problem	Post scores No problem /Mild problem	Pre scores Moderate to very severe problem	Post scores Moderate to very severe problem	Worsened	No change	Improved
Acid Regurgitation/Gas (Q1,2,22)					3(15%)	6(30%)	11(55%)
Q1 Belching /heart burn	14	19	6	1	3(15%)	9(45%)	8(40%)
Q2 Dysphagia	19	20	1	0	1(5%)	16(80%)	3(15%)
Q22 Excessive belching	17	19	3	1	0(0%)	14(70%)	6(30%)
Diarrhoea/incontinence (Q 8,11,12,13,14,21)					1(5%)	1(5%)	18(90%)
Q8 Pain or discomfort prior to a bowel motion	10	17	10	3	1(5%)	12(60%)	7(35%)
Q11 Loose stools	6	17	14	3	0(0%)	6(30%)	14(70%)
Q12 Incontinence	18	20	2	0	0(0%)	14(70%)	6(30%)
Q13 Urgency to empty bowel	3	13	17	7	2(10%)	5(25%)	13(65%)
Q14 Diarrhoea	10	17	10	3	1(5%)	6(30%)	13(65%)
Q21 Excessive gas and passing of wind	7	15	13	5	0(0%)	9(45%)	11(55%)
Constipation (Q9&10)					2(10%)	10(50%)	8(40%)
Q9 Difficulty with emptying the bowel	13	16	7	4	2(10%)	9(45%)	9(45%)
Q10 Constipation	17	18	3	2	4(20%)	9(45%)	7(35%)
Nausea/vomiting (Q15,17,18,19)					1(5%)	16(80%)	3(15%)
Q15 Loss of appetite	17	19	3	1	3(15%)	13(65%)	4(20%)
Q17 Sickness	20	20	0	0	0(0%)	19(95%)	1(5%)
Q18 Nausea	20	20	0	0	0(0%)	18(90%)	2(10%)
Q19 Vomiting	20	20	0	0	0(0%)	19(95%)	1(5%)
Epigastric pain/discomfort (3,4,5,6,7,16,20)					1(5%)	7(35%)	12(60%)
Abbreviations: SAGIS = Structured Assessment of Gastrointestinal Symptoms							

Supplementary Table 2. Participants' acceptance of information provided

	No	Yes	Comments

Was the diet taught in a way that you could easily understand it? (N=19)		100% (n=19)	▪ *It took some work and reading to get things on track*<br>▪ *The explanations made the following read through easier*
Do you think you would have been able to follow the diet if only written information was given with no oral explanation? (n=16)	57% (N=9)	43% (N=7)	▪ *I was glad to have the oral explanation because my concentration span lessens with my age.*<br>▪ *Yes I could have followed it but was better to have contact with a dietitian.*<br>▪ *I think it would have been quite daunting without an oral explanation.*<br>▪ *The oral explanation helped with motivation to do the diet.*

Supplementary Table 3. Participants' perception of following the low FODMAP diet

Do you think you spent more or less than your usual shopping<br>N = 13	No	Yes	Extra spent per week	N
	54%<br>N=7	46%<br>N=6	Participant indicated spending more but no dollar amount given	2
			$10-$15	2
			> $20 a week	2
Do you think you spent more or less time than you usual shopping<br>N=5	More time	Comments		
	N=5	I spent more time trying to find the right food I could eat and finding if it was safe to eat		
Were there any other resources that would have been helpful<br>N= 19	No	Yes	Comments	
	79%<br>N =15	21%<br>N = 4	▪ *FODMAP magazine - Australian. Added authenticity /proof that what I was experiencing was not uncommon.*<br>▪ *Knowing where to find low FODMAP products in the supermarket*	
What was the most difficult part of the diet to follow	Comment			N
	Missing high FODMAP foods			5
	Getting used to the change in diet/getting started			4
	Eating the gluten free bread – expensive and/or taste			3
	Entertaining or eating out			2
	Reading food labels			1
	Often there wasn't any food I could eat so was hungry several times			1

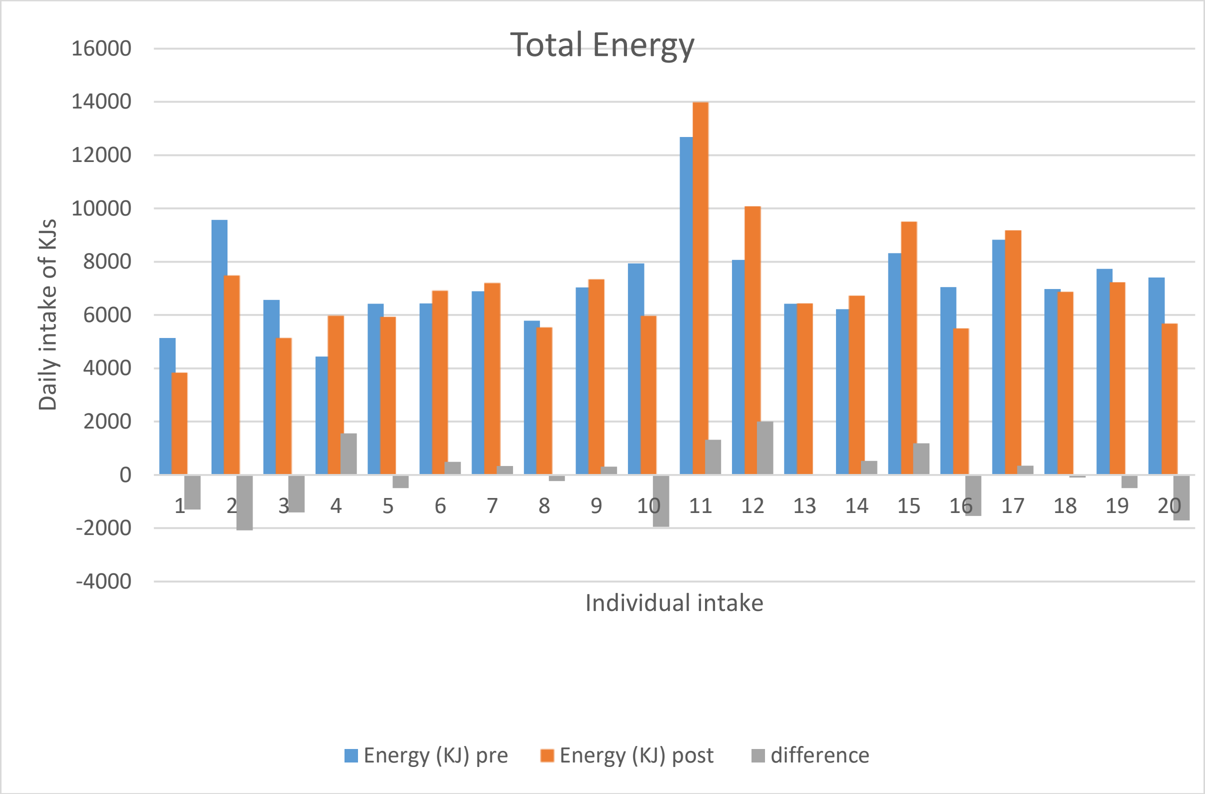

Supplementary Figure 1. Individual total energy (KJ) differences at baseline and follow up. Data represents the energy intake of each individual at baseline, follow up and the difference between intakes.

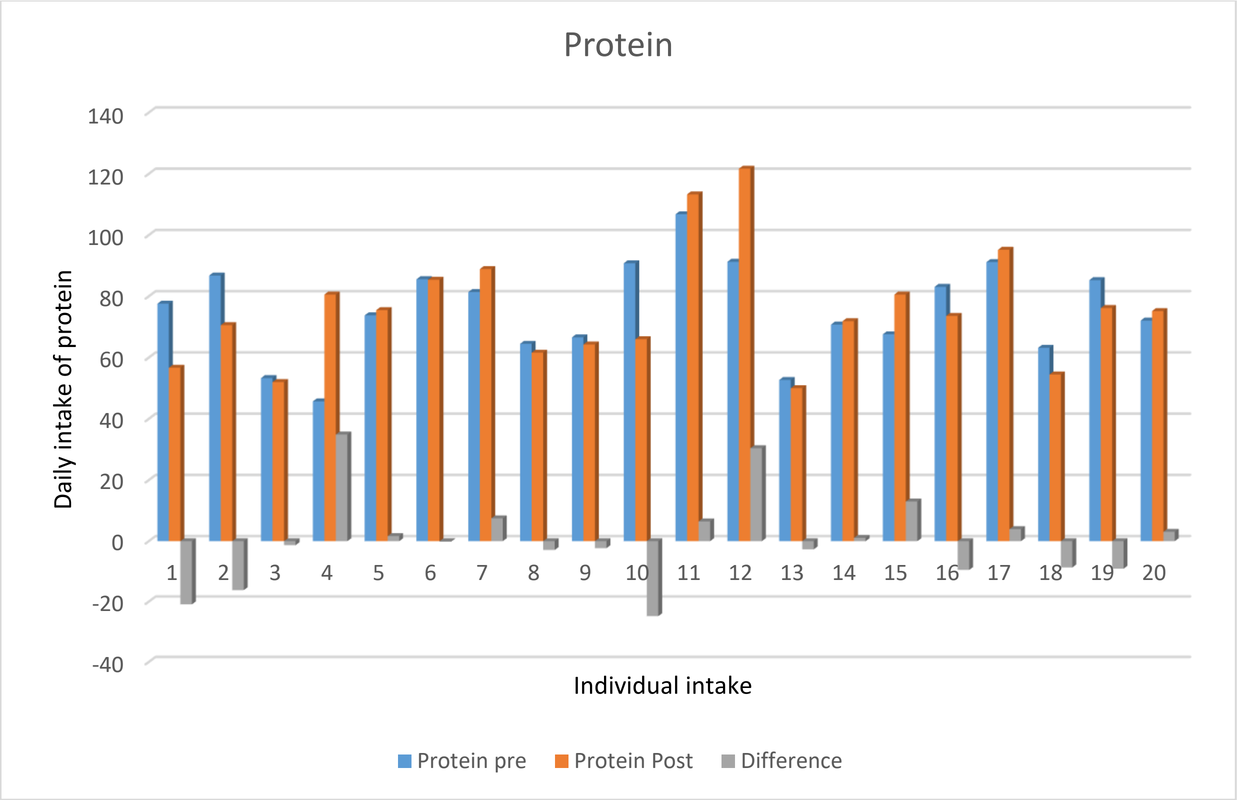

Supplementary Figure 2. Individual protein intake at baseline and follow up. Data represents the protein intake of each individual at baseline, follow up and the difference between intakes.

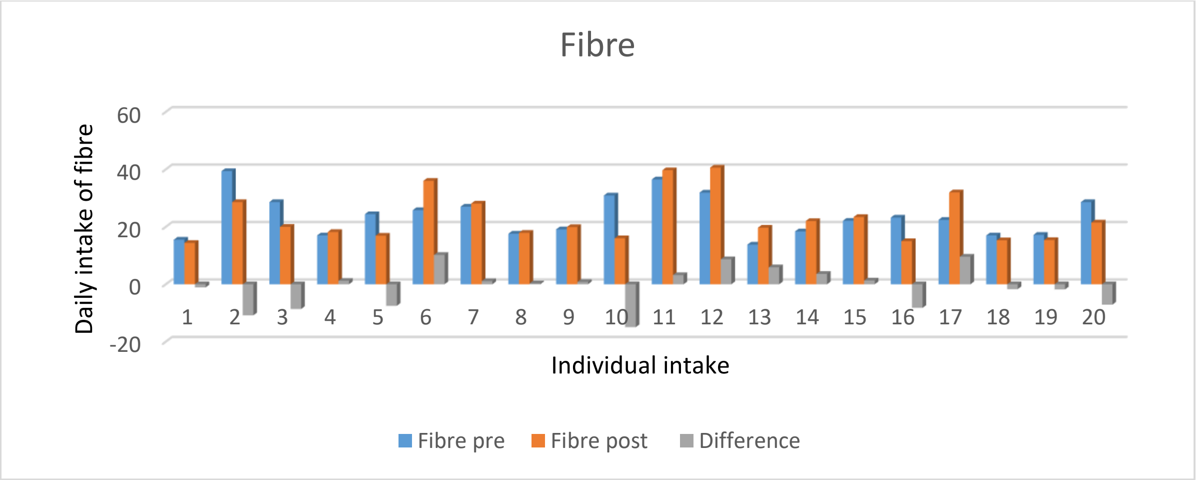

Supplementary Figure 3. Individual fibre intake at baseline and follow up. Data represents the fibre intake of each individual at baseline, follow up and the difference between intakes.
